# Homeostatic pulmonary microenvironment is responsible for alveolar macrophages resistance to endotoxin tolerance

**DOI:** 10.1186/cc10613

**Published:** 2012-03-20

**Authors:** F Philippart, C Fitting, B Misset, J Cavaillon

**Affiliations:** 1 Groupe Hospitalier Paris Saint Joseph, Paris, France; 2 Institut Pasteur de Paris, Paris, France

## Introduction

Endotoxin tolerance (ET) is a modification of immune response to a second challenge with lipopolysaccharide (LPS), which results in a decreased production of proinflammatory cytokines, and is considered partly responsible for the susceptibility to infectious processes in hospitalized patients [1]. We previously observed an absence of ET of alveolar macrophages (AM) to LPS in an *ex vivo *murine model of endotoxin tolerance [2]. We hypothesized that this singularity could be mediated by granulocyte-macrophage colony-stimulating factor (GM-CSF) (known to be predominantly produced by type II pneumocytes) and interferon-gamma (INFγ), two cytokines known to prevent the occurrence of ET [3]. The objectives were to confirm the absence of tolerance of AM to LPS and to assess the respective roles of GM-CSF and INFγ in this phenomenon and the cellular origin of INFγ.

## Methods

We used different wild-type mice strains (BALB/c, C57BL/6,129SV), and KO mice lacking different leukocytes subset rag2^-/-^, rag2gc^-/-^, cd3e^-/-^, μ^-/-^, il-15^-/- ^and Ja18^-/-^. We used an *ex vivo *model consisting of intravenous injection of LPS 20 hours prior to an *in vitro *stimulation of AM, peritoneal macrophages and monocytes with LPS. We pretreated the wild-type mice with anti-cytokines antibodies, and KO mice with B cells and NK cells adoptive transfer.

## Results

We confirmed the absence of AM tolerance to endotoxin in all the strain of wild-type mice. Inhibiting either GM-CSF or INFγ *in vivo *at homeostasis led to a decrease in TNF production by AM during the *in vitro *stimulation by LPS, suggesting the involvement of these cytokines in the prevention of tolerance within the lungs. The fact that AM from rag2^-/-^, rag2gc^-/-^, μ^-/- ^could be tolerated, the fact that adoptive transfer of B lymphocytes in these deficient mice restores the wild-type response, and the presence of INFγ mRNA in the lungs at homeostasis in wild-type mice and before and after adoptive B-lymphocyte transfer in KO mice demonstrated the involvement of these cells in the wild-type phenotype.

## Conclusion

We confirm the resistance of AM to endotoxin tolerance. Both GM-CSF and INFγ within the lung microenvironment at homeostasis are involved in this phenomenon. B lymphocytes play a key role in the local expression of INFγ.
